# Exploring Bioactive Polyphenolic Compounds in Food and Natural Real-World Samples II: Molecular Diversity, Functionality, and Future Directions

**DOI:** 10.3390/molecules31030537

**Published:** 2026-02-03

**Authors:** Francesco Cacciola, Katia Arena

**Affiliations:** Department of Chemical, Biological, Pharmaceutical and Environmental Sciences, Messina Institute of Technology, University of Messina, Viale G. Palatucci 13, 98168 Messina, Italy; arenak@unime.it

## 1. Introduction

Polyphenolic compounds have long been recognized as one of the most structurally diverse and biologically relevant classes of secondary metabolites in plant-derived foods and natural products [1]. Their importance extends far beyond their classical role as antioxidants, encompassing a broad spectrum of biological, technological, and ecological functions [2]. In food systems, polyphenols contribute to sensory attributes, oxidative stability, and shelf life; in biological systems, they interact with cellular pathways, enzymes, and microbial communities; and in broader sustainability frameworks, they represent key molecular targets for the valorization of agricultural resources and by-products [3]. Against this background, this Special Issue of Molecules entitled “Exploring Bioactive Polyphenolic Compounds in Food and Natural Real-World Samples II” was conceived as a continuation of a previous collection, with the explicit aim of advancing polyphenol research beyond simplified models and toward realistic matrices, scalable processes, and biologically meaningful contexts.

The motivation behind this Special Issue arises from a growing consensus within the scientific community that isolated-compound approaches alone do not full capture the functional properties of polyphenols. While reductionist studies remain indispensable for mechanistic understanding, there is an increasing need to investigate polyphenols as they occur in complex food matrices, plant tissues, and natural samples, where interactions with other constituents profoundly influence their chemical behavior and biological effects [4]. The contributions assembled in this Special Issue respond directly to this challenge by focusing on real-world samples, underutilized materials, and technologically relevant processes to provide a more holistic perspective on polyphenolic compounds and their applications.

## 2. Overview of Published Articles

Across the literature, a central and recurring theme is the sustainable sourcing and valorization of polyphenols from non-conventional or secondary materials [5]. Several contributions demonstrate that plant parts traditionally regarded as waste, such as oil press-cakes (Contribution 1), fruit husks (Contribution 2), and distillation residues (Contribution 3), can serve as rich reservoirs of phenolic compounds with significant bioactive potential. By combining detailed chemical profiling with functional evaluation, the authors of these studies challenge conventional perceptions of value within food and agro-industrial chains. For instance, the investigation of press-cake powders derived from oilseed processing illustrates how lipid-depleted matrices can retain concentrated phenolic fractions capable of exerting antioxidant and other biological effects. Similarly, the characterization of phenolics recovered from hydro-distillation by-products highlights the possibility of closing material loops within essential oil production systems. Collectively, these works align strongly with the principles of a circular economy, emphasizing resource efficiency, waste minimization, and the creation of added value through molecular-level insight.

Methodological innovation in extraction and processing constitutes another major pillar of this Special Issue. The efficient recovery of polyphenols from complex matrices remains a critical challenge, particularly when scalability, energy consumption, and compound stability must be considered simultaneously (Contribution 4). The application of microwave-assisted extraction, the optimization of drying conditions, and the development of scalable extraction protocols exemplify how process parameters can be fine-tuned to maximize phenolic yield while preserving chemical integrity. Importantly, these studies move beyond purely analytical optimization by addressing technological feasibility and process robustness, thereby narrowing the gap between laboratory research and industrial application. The explicit consideration of factors such as drying temperature and extraction scale underscores the sensitivity of polyphenolic compounds to processing conditions and reinforces the need for carefully designed workflows in both research and production settings.

High-level analytical characterization remains a foundational element of polyphenol research, and the contributions to this Special Issue showcase the power of advanced chromatographic and spectrometric techniques in unraveling complex phenolic profiles. Detailed qualitative and quantitative analyses enable not only the identification of well-established phenolic compounds but also the discovery of previously unreported molecules in wild or less-studied plant species. Comparative studies involving different species, growth conditions, or plant parts further reveal how genetic, environmental, and physiological factors shape polyphenol composition (Contribution 5). These findings underscore the vast and incompletely mapped chemical diversity of polyphenols in nature and highlight the importance of continued methodological refinement to ensure accurate, reproducible, and meaningful characterization.

Beyond compositional analysis, a significant number of contributions integrate chemical data with biological and functional assessments, reflecting an important shift toward analyzing structure–function relationships. Antioxidant activity, antiglycation effects, and metabolic modulation are evaluated using in vitro and ex vivo models, providing insight into the potential health relevance of polyphenol-rich materials. In particular, studies addressing oxidative stress and glycation processes in the context of hyperglycemia emphasize the relevance of polyphenols for metabolic health and chronic disease prevention (Contribution 6). These investigations demonstrate that polyphenols should not be regarded as passive radical scavengers but rather as dynamic molecular actors capable of influencing complex biochemical networks. By linking phenolic composition to functional outcomes, the chosen published works strengthen the scientific basis for the rational development of functional foods and nutraceutical ingredients.

One of the most contemporary and rapidly evolving aspects addressed in this Special Issue is the interaction between polyphenols and gut microbiota (Contribution 7). Growing evidence suggests that many polyphenols exert their biological effects indirectly, through the modulation of microbial composition and activity, as well as through the generation of bioactive metabolites. The investigation of polyphenol-rich extracts in relation to both healthy and diseased gut microbiota provides valuable insight into these complex interactions. Such studies highlight the bidirectional relationship between dietary polyphenols and microbial ecosystems, where polyphenols influence microbial functionality and, in turn, microbial metabolism shapes polyphenol bioavailability and activity. This emerging paradigm adds an additional layer of complexity to polyphenol research and encourages future research involving personalized nutrition and microbiota-targeted interventions.

Plant physiology and agronomic practices have also emerged as influential determinants of polyphenol content and composition. Preliminary investigations into elicitation strategies demonstrate that controlled stress conditions can stimulate phenolic accumulation in plant tissues, offering promising avenues for enhancing the functional value of agricultural products at the source (Contribution 8). Although exploratory, these studies suggest that polyphenol optimization need not be limited to post-harvest processing but can be integrated into cultivation strategies, thereby aligning agricultural practices with nutritional and functional objectives. Such approaches may prove particularly valuable in the context of sustainable agriculture and climate-resilient crop production.

From a broader perspective, the contributions to this Special Issue collectively illustrate the multidimensional nature of contemporary polyphenol research. Advances in analytical chemistry, processing technologies, biological evaluation, and system-level understanding are increasingly interconnected, and progress in one area often depends on developments in another. At the same time, the authors of these studies bring attention to persistent challenges that continue to shape the field. These include the need for standardized analytical protocols to facilitate comparability between studies, improved models for assessing bioaccessibility and metabolism, and greater integration of chemical and biological data to support evidence-based health claims.

Interdisciplinary collaboration emerges as a critical driver of progress in this area. Chemists, food scientists, biologists, nutritionists, and technologists must work together to translate molecular insights into practical applications that are safe, effective, and sustainable. Future research will likely benefit from the incorporation of multi-omics approaches, advanced computational tools, and in vivo models capable of capturing the complexity of polyphenol–matrix–host interactions. Such integrative strategies will be essential for moving beyond descriptive studies toward predictive and application-oriented frameworks.

## 3. Conclusions

This Special Issue titled “Exploring Bioactive Polyphenolic Compounds in Food and Natural Real-World Samples II” provides a comprehensive and timely overview of current research directions in this vibrant field. By emphasizing real-world matrices, sustainable sourcing, methodological rigor, and biological relevance, the collected contributions advance our understanding of polyphenolic compounds and their multifaceted roles in food systems and human health. The guest editors sincerely thank all the authors for their high-quality contributions and commitment to advancing research on polyphenols. We also express our gratitude to the reviewers for their thoughtful and constructive evaluations and to the Molecules editorial team for their professional support throughout the publication process. It is our hope that this Special Issue will serve as a valuable reference point and inspire further interdisciplinary research into the chemistry, functionality, and applications of bioactive polyphenolic compounds.
